# Effects of *Allium sativum* Stem Extract on Growth and Migration in Melanoma Cells through Inhibition of *VEGF, MMP-2*, and *MMP-9* Genes Expression

**DOI:** 10.3390/molecules27010021

**Published:** 2021-12-21

**Authors:** Da-Hye Gam, Jae-Hyun Park, Jun-Hee Kim, Dong-Ho Beak, Jin-Woo Kim

**Affiliations:** 1 Department of Food Science, Sun Moon University, Natural Science 118, 70 Sunmoon-ro 221, Tangjeong-myeon, Asan-si 336-708, Korea; ank7895@naver.com (D.-H.G.); gengihoo@naver.com (J.-H.P.); jun981014@naver.com (J.-H.K.); kk900600@naver.com (D.-H.B.); 2 FlexPro Biotechnology, Natural Science 128, 70 Sunmoon-ro 221, Tangjeong-myeon, Asan-si 336-708, Korea

**Keywords:** *Allium sativum* stem, anticancer, antimetastasis, *VEGF*, *MMP-2*, *MMP-9*, gallic acid

## Abstract

The present study investigated the effects of *Allium sativum* stem extract (ASE) on B16-F0 cell growth and metastasis. Evaluation of the effects of ASE on B16-F0 cells’ viability and migration showed that 0.5 mg/mL ASE inhibited B16-F0 cells’ growth by 30.2% and migration by 38.5%, which indicates that the ASE has anticancer and antimetastatic effects on B16-F0 cells. To study the anticancer and antimetastatic mechanism, mRNA levels of vascular endothelial growth factor (*VEGF*), matrix metalloproteinases-2 (*MMP-2*), and matrix metalloproteinases-9 (*MMP-9*) expressions were evaluated with reverse transcription polymerase chain reaction, and 0.25 and 0.5 mg/mL ASE was found to exert significant inhibition on mRNA expressions of *VEGF*, *MMP-2*, and *MMP-9* in B16-F0 cells. Thus, ASE reduce extracellular matrix degradation through inhibitions of expression of *MMP-2* and *MMP-9,* and also showed an angiogenesis inhibitory effect through reduction of *VEGF* expression. High-performance liquid chromatography analysis showed that among various polyphenols, gallic acid (2.1 mg/g) was a major compound of ASE. Overall, our results demonstrated that ASE inhibited the growth and migration of B16-F0 cells through downregulation of the *VEGF, MMP-2*, and *MMP-9* genes expression, which indicates ASE could be applied for the prevention and treatment of melanoma.

## 1. Introduction

Skin cancer refers to a form of malignancy that can occur in the cells constituting the skin tissue and cause abnormal growth of skin cells [1]. Its typical types include squamous cell carcinoma, basal cell carcinoma, and melanoma. The first two skin cancers are grouped as non-melanoma skin cancers in the outer and middle layers of skin, while other unusual types of skin cancer include Merkel cell carcinomas and dermatofibrosarcoma protuberans in the deeper layer of the skin [2,3]. According to the World Health Organization (WHO), in 2018, 287,723 cases of melanoma skin cancer and 1,042,056 of non-melanoma skin cancer were diagnosed globally. In 2018, over 4 million people are diagnosed with skin cancer every year worldwide, and 60,712 people died of melanoma skin cancer and 65,155 of non-melanoma skin cancer. Incidence rates of melanoma skin cancer rose by 44% between 2008 and 2018, with deaths increasing by 32% [4,5]. On the other hand, in Korea, skin cancer has a low rate of incidence, comprising only 1.8% of all cancer cases [6]. However, according to Statistics Korea’s data, in 2020, the number of patients with skin cancer increased from 19,236 in 2016 to 27,211 (a 41.5% increase) and is showing a rapidly increasing trend [7]. In particular, the number of patients with malignant melanoma, which has the worst prognosis of all the skin cancers, increased by 30% [8]. Melanoma is malignant cancer that originates in the melanocytes and is recognized as an illness requiring active prevention and treatment through early diagnosis because of its high propensity for metastasizing to the bones, heart, and brain, where cancer cells normally do not metastasize [9]. However, melanoma has no early symptoms, making early detection difficult, and in many cases, it has already metastasized by the time of diagnosis; thus, the development of effective treatments to inhibit a melanoma’s growth and metastasis is essential [10].

Metastasis is a multistep process during which cancer cells, responding to different intrinsic and extrinsic stimuli, detach from the primary cancer tissues and invade surrounding tissues and blood or lymphatic vessels [11]. During metastasis, cancer cells detach from the primary site and pass through the basement membrane of the vascular endothelial cells to reach other tissues by traveling through the blood and lymphatic vessels [12,13]. Once they reach other tissues, cancer cells form new blood vessels that supply oxygen and nutrients so that they can continue their proliferation [14]. To pass through the basement membrane of the vascular endothelial cells, it is essential to break down type IV collagen, one of the main components of the basement membrane [15]. Cancer cells secrete matrix metalloproteinases (MMPs), such as *MMP-2* and *MMP-9*, for breaking down type IV collagen and promoting their growth and migration [16,17]. During this process, vascular endothelial growth factor (*VEGF*), an angiogenesis gene, plays a pivotal role in promoting the expressions of antimetasis *MMP-2* and *MMP-9* by binding to *VEGF* receptor-2 (*VEGFR-2*) and signaling extracellular signal-regulated protein kinases 1/2 (*ERK1/2*) and phosphoinositide 3-kinase (*PI3K*)/Akt to promote survival, growth, migration, invasion, and angiogenesis of melanoma cells [18,19,20]. According to Boocock et al., the expression of *VEGF* in ovarian carcinoma significantly enhanced MMP secretion has been associated with cancer growth and invasion. High levels of *VEGF* were found in serum or plasma and ascites of ovarian cancer patients, and a strong correlation between ascites volume and *VEGF* levels was reported in several experimental models [21]. As a result, inhibitors of *VEGF* activity reduced the formation of cancer and metastasis in abdominal and human ovarian cancer models [22,23]. Therefore, it is anticipated that the discovery of novel anticancer compounds that inhibit the expressions of *MMP-2*, and *MMP-9* by *VEGF* will contribute toward overcoming skin cancer via the regulation of melanoma growth and metastasis [24].

Garlic (*Allium sativum*) is a bulbous perennial plant of the *Allium* genus of the *Liliaceae* family whose main component, allicin, is responsible for the typical garlicky smell [25]. Allicin also has remarkable sterilization and antibacterial properties against bacteria and fungi and shows many health benefits owing to its anti-hypertensive and anti-mutagenic effects [26]. The reason that allicin is being spotlighted is because of its powerful anticancer properties, which inhibit cellular mutation and reduce cancer size, and since it is especially effective in preventing cancers, including gastric and colon cancers as well as prostate and uterine cancers [27]. According to the Food and Agriculture Organization of United Nations Statistics (FAOSTAT), world garlic production reached a peak of more than 28 million tons in 2017, rising from 11 million in 2000 [28]. Only the bulbous portion of the garlic is used and, consequently, the stems are treated as a by-product [29]. Due to the increase in garlic cultivation, the number of discarded garlic stems has also rapidly increased, resulting in enormous disposal costs and environmental pollution problems [30].

Research on food-derived active ingredients for cancer prevention as well as treatment is growing due to the relatively low or no toxicity. Several compounds derived from naturals have demonstrable anticancer effects by multiple mechanisms, including enhanced inducing apoptosis of cancer cells, perturbing cell cycle progression, and inhibiting metastasis, and angiogenesis. For example, previous studies on anticancer drugs showed that garlic has reduced the proliferation of various types of cancer, such as skin, colon, stomach, lungs, breast, and prostate, by reducing carcinogenesis, including mutations, cell proliferation, and differentiation [31]. Compounds contained in garlic, including allicin, ajoene, diallyl sulfide, diallyl disulfide, and diallyl trisulfide contribute to the distinct flavors and odors and the prevention of various diseases and cancers [32]. However, there have been no studies on the analysis of bioactive compounds in *A. sativum* stem and their effects on cancer growth and metastasis, so there is a high need for analyzing the ingredients of *A. sativum* stem and evaluation of anticancer effects. The aim of this study was to evaluate the effects of *A. sativum* stem extract (ASE) on melanoma cell growth and metastasis inhibition. We identified the main component in ASE and investigated the mechanism underlying the antimetastasis effect in regulating the MMP signaling of extract. Overall, our results provided valuable information related to the use of *A. sativum* stem in skin-cancer drug development.

## 2. Results and Discussion

### 2.1. Effect of ASE on Melanoma Cell Growth

**Figure 1:**
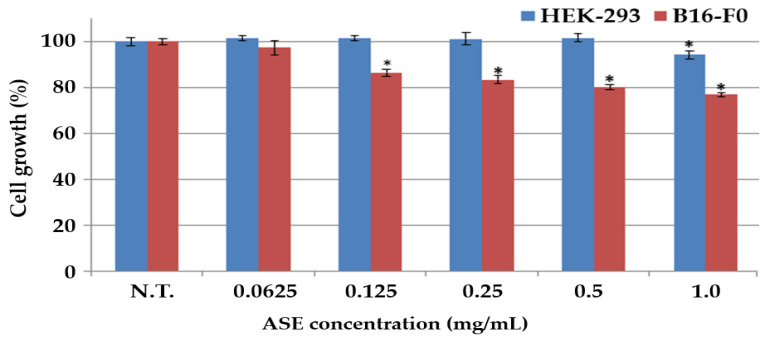
Comparison of cytotoxic effects of ASE on melanoma cells (B16-F0) and normal cells (HEK-293). The cell growth of the control group with the nontreated group (N.T.) at 24 h was represented as 100%. Results are mean ± standard deviation (*n* = 3). The statistical analysis of the data was carried out by use of a Student’s *t*-test. Values are statistically significant at * *p* < 0.05 vs. respective control group.

To evaluate the anticancer effect of ASE on melanoma, both normal kidney cells (HEK-293) and melanoma cells (B16-F0) were treated with different concentrations of ASE, and their cell growth was compared (Figure 1). Both HEK-293 and B16-F0 cells showed a decrease in their cell growth when treated with higher concentrations of ASE (*p* < 0.05). Furthermore, the effect of ASE on B16-F0 cell growth was evaluated. Up to a concentration of 0.5 mg/mL, cell growth of HEK-293 showed no difference when compared with that of the control group of HEK-293 (*p* > 0.05), but concentrations from 0.125 mg/mL and above showed significant reductions in cell growth of B16-F0, confirming that ASE is nontoxic to the normal cells at a concentration of ≤ 0.5 mg/mL while inhibition melanoma cell growth at a concentration of 0.125 mg/mL (*p* < 0.05). As a result of evaluating the effect of ASE concentrations on B16-F0 cell growth, it was seen that the growth of the melanoma cells dropped significantly at a concentration of ≤ 0.125 mg/mL, unlike normal cell, leading to the conclusion that ASE effectively inhibits the growth of HEK-293 without inhibiting the growth of B16-F0. 

An et al. reported that viability of BxPC-3, AsPC-1, and MIAPaCa-2 pancreatic cancer cells decreased below 80% when treated with a water extract of *O. Obtriangulata*, resulting in an anticancer effect [33]. Our experiments also showed that the inhibitory effect against melanoma cell growth was decreased by 80% when treated with an ASE. This decreasing effect is due to the occurrence of bioactive compounds in ASE that can inhibit melanoma cell growth [34]. Previous studies reported that ingredients in natural products such as polyphenols can significantly decrease the growth of cancer cells [35]. In particular, Zhao et al. reported that polyphenols such as gallic acid, ferulic acid, anthocyanin, and quercetin at high concentrations in *Allium* vegetables can significantly inhibit the growth of cancer cells, including breast cancer, gastric cancer, stomach cancer, and skin cancer [36]. A recent study reported that garlic significantly inhibits the growth of human blood cancer cells (HL-60) in an addition of 8 mg/mL of extract [37]. In conclusion, ASE inhibited the growth of B16-F0 cells at concentrations of ≥ 0.125 mg/mL, whereas the growth of HEK-293 cells was inhibited at concentrations of ≥ 1.0 mg/mL. This showed that at concentrations of 0.125–0.5 mg/mL ASE is safe for human cell treatment and possesses an anticancer effect against melanoma cells. These data indicate that ASE has selective cytotoxic effects on melanoma cells, and this selectivity is thought to be a great advantage of the ASE for therapeutic or preventive use in cancer treatment.

### 2.2. Effect of ASE on Melanoma Cell Migration

**Figure 2:**
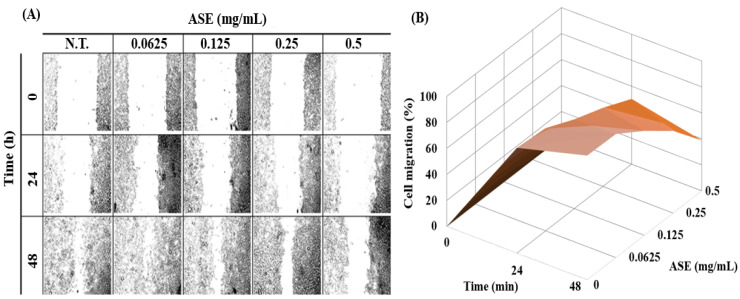
Effect of ASE on the B16-F0 cell migration. Migration rates were quantitatively analyzed by calculating the difference between wound area of ASE-treated and control groups at 0, 24, and 48 h. Results are expressed as mean ± standard deviation (*n* = 3), (**A**) Images of wound-healing assays. All the images show the progress of wound closure on scratch wounded B16-F0 cells. (**B**) 3-D graph showing the effect of ASE concentration and wound healing time on B16-F0 cell migration. The statistical analysis of the data was carried out by use of a Student’s *t*-test.

Cell migration significantly decreased, depending on both the concentration of ASE and treatment duration, demonstrating that ASE concentration and treatment durations can both act as primary variables of B16-F0 cell migration (*p* < 0.05) (Figure 2A). The control group, which was not treated with ASE, showed 37.1% cell migration after 24 h, but it significantly decreased to 26.1% when treated with 0.5 mg/mL of ASE. This confirmed that ASE effectively inhibits B16-F0 cell migration. In addition, while the control group showed 95.5% cell migration after 48 h, cell migration significantly decreased to 43.7% when the B16-F0 cells were treated with 0.5 mg/mL of ASE, demonstrating that the inhibitory effect of cell migration was increased compared to 24 h (Figure 2B). When compared to Su’s study on the anticancer effect of *Hibiscus* hot-water extract against melanoma cell migration in which B16-F0 cells treated with 5.23 mg/mL of *Hibiscus* hot-water extract for 48 h showed 50.0% migration, the present study showed lower B16-F0 cell migration, proving that ASE is more effective in inhibiting melanoma cell migration of ASE compared to other extracts [38]. Cell migration is essential for cancer cells to form blood vessels in angiogenesis and is necessary for cancer growth and metastasis [39]. Research on cell migration in cancer research is of great interest because the main cause of death in cancer patients is highly related to metastatic progression. Thus, there is an urgent demand to develop a new therapeutic agent for cancer metastasis [40,41]. Taken together, these results suggested that ASE could act as an effective treatment for melanoma suppression by inhibiting cell proliferation, migration, and invasion in B16-F0 cells.

### 2.3. mRNA Expressions of VEGF, MMP-2, and MMP-9

**Figure 3:**
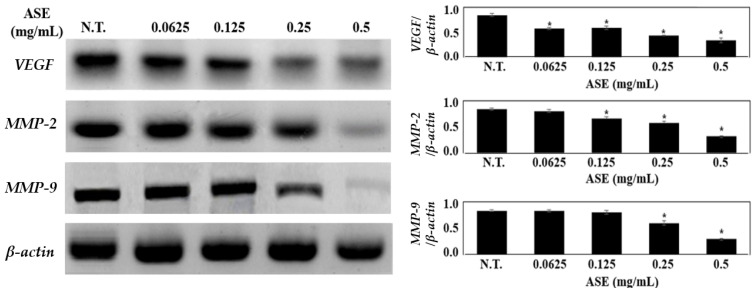
The effect of ASE on expressions of *VEGF*, *MMP-2*, and *MMP-9* mRNA. *MMP-2* and *MMP-9* which are members of the MMP family and implicated in cancer cell migration, invasion, and metastasis in various cancers. *VEGF* is a major angiogenic growth gene found in cancer cells. *β*-*actin* was used as an internal control. Each result in the graph is presented as the mean ± standard deviation based on band intensity for each group (*n* = 3). Values are statistically significant at * *p* < 0.05 vs. respective control group.

ASE inhibited the expressions of *MMP-2* and *MMP-9*, which are enzymes that hydrolyze a type IV collagen of basement membrane, in a concentration-dependent manner (Figure 3). A significant decrease in *MMP-2* and *MMP-9* expressions is observed in the experimental groups treated with 0.25 mg/mL and 0.5 mg/mL ASE when compared to the control group (*p* < 0.05). ASE also inhibited the expression of *VEGF* in a concentration-dependent manner, and in particular, its expression was suppressed by 58.6% in the experimental group treated with 0.5 mg/mL of ASE when compared to that of the control group. A previous study by Shin et al. indicated that metastasis is inhibited when *MMP-2* and *MMP-9* are downregulated by black garlic extract with a concentration of 0.5 mg/mL in the human gastric cancer cells, and Barbieri et al. reported that *Ganoderma Iucidum* extract decreased the *MMP-2* and *MMP-9* mediated breakdown of type IV collagen and was effective in impeding breast cancers’ metastasis and invasion by inhibiting colon cancer cells’ migration [42,43]. Furthermore, Kim et al. showed that decreased *VEGF* expression in oral squamous cell carcinoma that was xenografted to mice resulted in angiogenesis inhibition and cancer volume reduction, proving that *VEGF* is an important modulator of cancer cell growth and metastasis [44].

Abnormal proliferation, invasion, and metastasis are the basic biological characteristics of malignant cancers [45]. These are not only associated with the enhanced invasiveness and the decreased adhesion of cancer cells, but also are closely related to angiogenesis, ECM degradation, and interstitial remodeling [46]. Thus, anticancer and antimetastasis effects by ASE induce the downregulation of type IV collagen-targeted mRNAs, which cause degradation of the basement membrane. Our results support previous MTT and cell migration results indicating that ASE may decrease *VEGF, MMP-2*, and *MMP-9* expressions, thereby inhibiting the proliferation of melanoma cells.

### 2.4. Identification of Gallic Acid in ASE

**Figure 4:**
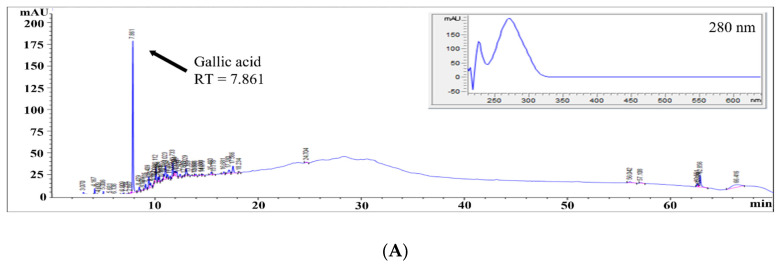

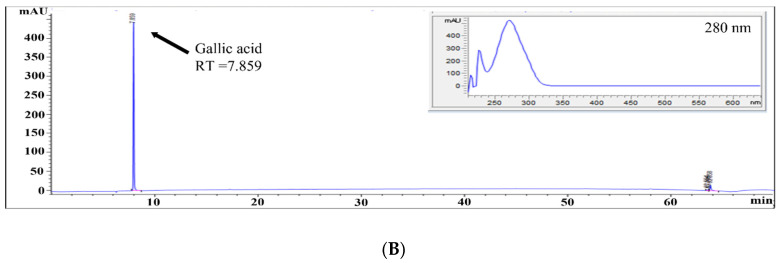
HPLC chromatograms for quantitative and qualitative analysis for the determination of gallic acid concentration in ASE at 280 nm. (**A**) Chromatogram and DAD spectrum (190–640 nm) of gallic acid from ASE. (**B**) Chromatogram and DAD spectrum (190–640 nm) of gallic acid standard (0.1 mg/mL).

The previous experiments confirmed the anticancer and antimetastasis effects of ASE on B16-F0 cells through cell growth inhibition and mRNA expression tests. Next, high-performance liquid chromatography (HPLC) analysis was performed to identify the active ingredients in ASE that showed anticancer and antimetastasis effects in previous studies. In extract analysis using HPLC, the main peaks of ASE were distributed at 5.512–20.78 min, and the highest peak was confirmed at a retention time (RT) of 7.861 min. The highest peak of ASE had the same RT as the gallic acid standard, and the maximum absorption wavelength of the diode array detector (DAD) spectrum was 280 nm and the pattern of the spectrum was the same (Figure 4). The amount of gallic acid present in the ASE was determined through a standard calibration curve using gallic acid (5.0–100.0 mg/mL). A standard calibration curve was constructed by plotting the peak areas against concentration, and the linear regression equations were applied to calculate the concentration of gallic acid in ASE. The gallic acid standard calibration curve shows good linearity between concentrations and the peak area, with a correlation coefficient (R^2^) of 0.998. After calculation, the amount of gallic acid present in the ASE was found to be 2.1 mg/g, which is 4.5 times higher than that obtained in Jang et al.’s study (0.46 mg/g of gallic acid) on *Cornus officinalis* hot-water extract [47].

Peroxidation of proteins, lipids, RNA, and DNA affects cellular function, thereby increasing the risk of cardiovascular disease and cancer, and accelerating the process of aging [48]. Gallic acid is a representative polyphenol that is reported to have alleviating effects on cellular DNA injury resulting from oxidative stress and is known to have many physiological uses owing to its antioxidant, antibacterial, anticancer, and anti-hypertension properties [49]. In addition, gallic acid is a representative polyphenol that is reported to have alleviating effects on cellular DNA injury resulting from oxidative stress and is known to have many physiological uses owing to its antioxidant, antibacterial, anticancer, and antihypertension properties. Previous studies have demonstrated that gallic acid is capable of selectively inducing apoptosis in various cancer cells, including HeLa, HL-60RG, dRLh-84, and PLC/PRF/5 [50]. Moreover, Kahkeshanithat et al. reported that gallic acid regulates the cell cycle-related proteins such as cyclin A, cyclin D1, and cyclin E, and slows down the cell division by inhibition of CDK and induction of the p27KIP [51]. Collectively, these data suggest that gallic acid, the main ingredient of ASE, contributes synergistically to the anticancer and antimetastasis effects on B16-F0.

## 3. Materials and Methods

### 3.1. Materials and Reagents

*A. sativum* stem was purchased from Nonghyup mart (Seoul, Korea) and stored in a refrigerator at −5 °C. Used in cell culture, Dulbecco’s modified Eagle medium (DMEM), fetal bovine serum (FBS), and trypsin–EDTA were purchased from Gibco-BRL Co., Ltd. (Gaithersburg, MD, USA). We obtained 3-[4,5-dimethylthiazol-2-yl]-2,5-diphenyl tetrazolium bromide (MTT) and dimethyl sulfoxide (DMSO) from Sigma-Aldrich Co., Ltd. (St. Louis, MO, USA). Acetonitrile and acetic acid for HPLC grade were purchased from Thermo Fisher Sci., Inc. (Waltham, MA, USA). All other chemicals used in this experiment were Sigma-Aldrich’s analytical grade unless otherwise noted.

### 3.2. Preparation of Extract

Prior to the experiment, the *A. sativum* stem was dried using the gravity convection dry oven (FC 49, Lab House Co., Seoul, Korea) for 24 h at 60 °C and powdered below 0.42 mm using a grinder (HMF-3000S, Hanil Co., Wonju, Korea). We placed 1 g of powdered *A. sativum* stem into a pressure vessel with 10 mL of the solvent and mixed it using a vortex mixer (VM-10, Daihan Sci. Co., Wonju, Korea) for 1 min. Ultrasound-assisted extraction (UAE) was conducted using an ultrasound device (SD-250H, Mujigae Co., Seoul, Korea). After UAE, the supernatant was separated from the mixture at 10,000 rpm for 10 min using a centrifuge (1236R, Labogene Co., Daejeon, Korea). Each supernatant was filtered through a 0.45 µm syringe filter (Hyundai Micro Co., Seoul, Korea) and then used for analysis.

### 3.3. Cell Culture

Human melanoma cells (B16-F0) and kidney cells (HEK-293) were purchased from the Korean Cell Line Bank (KCLB, Seoul, Korea). The cells were cultured in a DMEM medium with 10% FBS and 1% penicillin at 37.0 °C in a 5.0% CO_2_ incubator (MCO-5AC, Sanyo Co., Ltd., Tokyo, Japan) where they grew as monolayer cultures on the bottom of the 50 cm^2^ flasks. To subculture the cells, the DMEM medium was removed, 500 µL of trypsin-EDTA was then added, and the flasks were maintained in the incubator with a 5.0% CO_2_ incubator for 5 min. After the cells had detached from the bottom layer of flasks by trypsin-EDTA treatment, they were transferred to 50 cm^2^ flasks for sustainable growth. DMEM medium was exchanged once needed until cells reached 80–90% confluency.

### 3.4. MTT Assays

The effects of ASE on the growth of B16-F0 and HEK-293 cells were determined by the MTT assay [52]. Cells were seeded at 1×10^6^ cells/well in a 96-well plate and then treated with different concentrations of ASE (0.0-1.0 mg/mL) for 24 h. The control group was treated with the same volume of DMEM medium. A total of 100 µL MTT solution (0.25 mg/mL) at 4 h was then added to each well. After the completion of incubation, the MTT solution was removed and 500 µL DMSO was added to dissolve formazan crystals. The extent of reduction of MTT to formazan within the cells was calculated by measuring the absorption at 570 nm using a microplate reader (AMR-100, Allsheng Co., Ltd., Seoul, Korea) and data were collected for each three replicates and used to calculate the means and the standard deviations. The cell growth was calculated according to the below.

(1) $$\mathrm{Cell}\;\mathrm{growth}\;\left(\%\right)=\left\{1-\frac{\mathrm{Abs}\;\left(\mathrm{sample}\right)}{\mathrm{Abs}\;\left(\mathrm{control}\right)}\right\}\times 100$$

### 3.5. Wound Healing Assay

Cell migration ability was determined by wound healing assay [53]. B16-F0 cells were seeded at 1 × 10^5^ cells/well in a 96-well plate and maintained culture for 48 h. A vertical wound was made by scratch in cells using a 200 µL pipette tip in the vertical direction of the center of the wells. The cells were washed with PBS and cultured with 0.0–0.5 mg/mL ASE for 48 h to observe a decrease in wound area. As a control group, DMEM containing 0.25% DMSO without adding an extract was used. Images of migrated cells were taken using a digital camera connected to an optical microscope (Anton Paar Co., Ltd., Graz, Austria) to observe the closure of the wound area at 0, 24, and 48 h. Then, the reduction of the wound area of B16-F0 cells was measured using ImageJ software (National Institutes of Health, Bethesda, MD, USA). Cell migration rate was calculated as the percentage of the wound area at 48 h compared to the initial wound area at 0 h.

### 3.6. Reverse Transcription Polymerase Chain Reaction (RT-PCR)

RT-PCR was performed to determine the mRNA level of *VEGF*, *MMP-2*, and *MMP-9* in B16-F0 cells [54]. B16-F0 cells were cultured in a 24-well plate, each well containing 1.0 × 10^6^ cells. After 24 h, the cells were then cultured with 0.0–0.5 mg/mL ASE for 24 h. After collecting the cells, the total RNA was extracted from cells using AccuPrep^®^ universal RNA extraction kit (Bioneer Co., Daejeon, Korea) and quantified using NanoDrop™ 2000c spectrophotometer (Thermo Fisher Sci., Inc. Waltham, MA, USA). Total RNA (0.5 μg) was reverse transcribed into cDNA using amfiRivert cDNA synthesis platinum master mix (GenDEPOT Co., Katy, TX, USA) at 95 °C for 1 min, 50 °C for 30 min, and 75 °C for 4 min according to the manufacturer’s protocol. The cDNA was amplified with each primer such as *VEGF*, *MMP-2*, *MMP-9*, and *β*-*actin* (Table 1). The PCR conditions were performed with an initial denaturation at 94 °C for 5 min, followed by 30 cycles of 5 s at 95 °C, 30 s at 60 °C (*VEGF*) or 30 s at 59 °C (*MMP-2*) or 1 min at 54 °C (*MMP-9*), and 30 s at 72 °C. Each PCR product was electrophoresed on 1.0% agarose gel and visualized by using Gel Doc TM XR+system and quantity one software (Bio-Rad Co., Hercules, CA, USA). The abbreviations and main functions of the main genes used in this experiment are shown in Table 2.

### 3.7. HPLC Analysis

Quantitative and qualitative analysis of polyphenols in ASE was carried out by HPLC (Agilent, Palo Alto, CA, USA) equipped with a Zorbax SB C18 column (4.6 × 150 mm, Osaka Soda Co., Ltd., Osaka, Japan) and a DAD. The elution mobile phases were (A): 1.0% acetic acid and (B): 99.9% acetonitrile. The elution conditions for polyphenols were as follows; 0–5 min, 0.0–15.0% B; 5–50 min, 15–50% B; 50–60 min, 50–100% B; 60–64 min, 100–0.0% B. The flow rate was kept constant throughout the analysis at 0.5 mL/min, the injection volume of samples was 10 µL, and the wavelength range for absorption spectrum analysis was 190 to 640 nm. Identification of polyphenols was performed by comparing retention time and UV absorption spectra with those of standards. The quantity and quality analysis of the individual polyphenol in the ASE was determined based on a standard curve prepared for each standard compound under the same elution conditions as those used for the elution of the polyphenols in the ASE.

### 3.8. Statistical Analysis

All the data represent three independent experiments, and data were analyzed with Microsoft Excel 2010 (Microsoft Co., Redmond, WA, USA). Results were expressed as mean ± standard deviation (SD) of three independent experiments. Statistical significance between groups was assessed by unpaired Student’s *t*-test or, when necessary, by one-way analysis of variance. A *p* value < 0.05 was considered statistically significant.

## 4. Conclusions

In this study, the inhibitory effect of ASE on melanoma cell growth and metastasis was observed, and its anticancer mechanism was confirmed through the evaluations of B16-F0 cell growth inhibition and expression of major genes related to anticancer. Treatment with an ASE concentration of 0.125–0.5 mg/mL did not affect the cell growth of normal kidney cells, whereas B16-F0 had a decrease in cell growth at concentrations above 0.125 mg, resulting in a selective anticancer effect of only B16-F0 in the range of 0.125–0.5 mg/mL. Cell migration, a major factor related to cancer metastasis, was tested, and B16-F0 cell migration was significantly inhibited by 58.7% when treated with ASE of 0.5 mg/mL, confirming its effects in preventing melanoma metastasis. To control cancer growth, inhibition of the migration of the cancer cells is one of the essential strategies. During cancer metastasis, cancer cells migrate into neighboring healthy tissues, contributing to cancer development. Therefore, ASE could contribute to hindering growth and metastasis in melanoma.

Based on these results, the effect of ASE on the expressions of *VEGF*, *MMP-2*, and *MMP-9*, the major genes related to an anticancer effect, was evaluated and its metastasis-inhibiting effect at the genetic level was confirmed. This study confirmed that ASE downregulates the expressions of the mRNA of *MMP-2* and *MMP-9*, the genes related to enzymes that break down a basement membrane during metastasis and inhibit angiogenesis, which is promoted by *VEGF*. Thus, it is expected that ASE has an anticancer effect on melanoma and an inhibiting effect on melanoma invasion and metastasis through the reduction of the expressions of *VEGF*, *MMP-2*, and *MMP-9*, which are secreted during metastasis. The presence of active ingredients was also confirmed by HPLC analysis, and gallic acid in the ASE was measured at 2.1 mg/g. Gallic acid has been proven in previous studies to induced apoptosis as well as cytotoxicity or necrosis in several cancer cells, and the anticancer effect of ASE revealed in this study can be expected to originate from gallic acid in ASE. In conclusion, the present study suggests that ASE could be recommended as a preventive and therapeutic agent, based on its anticancer and antimetastasis properties. Based on the above results, further research on ASE’s potential in the prevention and treatment of melanoma through animal models and clinical trials will be needed in the future for the utilization of garlic stem for a pharmaceutical material.

## Figures and Tables

**Table 1:** List of primers used to determine gene expressions of *VEGF*, *MMP-2*, and *MMP-9* using RT-PCR. The sequence of designed primers for each gene is shown as forward and reverse.

Primer	Forward (5’–3’)	Reverse (5’–3’)	Size (bp)
*VEGF*	GCAGAATCATCACGAAGTGG	GCATGGTGATGTTGGACTCC	169
*MMP-2*	CAGCCTGGGACTGCCCCCTGAT	CAGGCCCCTCCGGGTCCTTCTC	400
*MMP-9*	AGTTTGGTGTCGCGGAGCAC	TACATGAGCGCTTCCGGCAC	754
*β-actin*	AGCACAGAGCCTCGCCTTT	CTTAATGTCACGCACGATTTCC	697

**Table 2:** Comparison of major gene expressions related to cell migration, proliferation, and enhancing vascular permeability. List of the abbreviations and functions of main genes comparing expressions in this experiment.

Genes	Functions	Abb.
Vascular endothelial growth factor	Specific growth factor for angiogenesis. A crucial factor of angiogenesis in tumor growth and metastasis.	*VEGF*
Matrix metalloproteinases-2	Degradation of gelatin, type IV collagen, and some bioactive molecules, such as growth factor-binding proteins receptors.	*MMP-2*
Matrix metalloproteinases-9	Degradation of type IV collagen, proteoglycan core protein, and elastin.	*MMP-9*
Matrix metalloproteinases	metalloproteinases capable of degrading all components of the extracellular matrix.	*MMPs*
Vascular endothelial growth factor receptor-2	Activation of *VEGF*-stimulated signal transduction including endothelial cell survival, migration, proliferation, enhancing permeability.	*VEGFR-2*
Phosphoinositide 3-kinase	Regulation of various cell functions including cell proliferation, apoptosis, tumor growth, and angiogenesis by Akt downstream.	*PI3K*
Extracellular signal-regulated protein kinases	Important messenger for extracellular and intracellular signals, which serve a vital role in processes, including proliferation, differentiation, cytoskeleton construction, and cellular senescence. Involvement in *VEGF*-C upregulation by inducing IGF-1.	*ERK1/2*

## Data Availability

No new data were created or analyzed in this study. Data sharing does not apply to this article.

## Sample Availability

Samples of the compounds are not available from the authors.
